# Chemotherapy-induced changes of cerebral activity in resting-state functional magnetic resonance imaging and cerebral white matter in diffusion tensor imaging

**DOI:** 10.18632/oncotarget.18111

**Published:** 2017-05-23

**Authors:** Caiqin Mo, Hailong Lin, Fangmeng Fu, Lin Lin, Jie Zhang, Meng Huang, Chuan Wang, Yunjing Xue, Qing Duan, Weiwen Lin, Xiangjin Chen

**Affiliations:** ^1^ Department of Breast Surgery, Affiliated Union Hospital of Fujian Medical University, Fuzhou, China; ^2^ Department of Thyroid and Breast Surgery, First Affiliated Hospital of Fujian Medical University, Fuzhou, China; ^3^ Radiology Department, Affiliated Union Hospital of Fujian Medical University, Fuzhou, China; ^4^ Fujian Center for Disease Control and Prevention, Fuzhou, China

**Keywords:** breast cancer, chemotherapy, cognitive disorder, resting-state functional magnetic resonance imaging, diffusion tensor imaging

## Abstract

While chemotherapy related cognitive disorder has been described in many studies, but we still lack relatively reliable and objective diagnostic tools, and there are few similar studies in Asian patients. We recruited Asian breast cancer patients to perform a cohort study to uncover chemotherapy related cognitive disorder by using resting-state functioning magnetic resonance imaging (RS-fMRI) and magnetic resonance diffusion tensor imaging (DTI) combined with neuropsychologic assessments. This is the first prospective study which combines RS-fMRI and DTI to detect chemotherapy related cognitive disorder. The neuropsychologic tests and MRI were performed before and after the chemotherapy. The healthy controls were tested at matched times. The chemotherapy-treated group performed worse on memory and we found significant changes in the cerebellum, right orbitofrontal area, right middle and superior temporal gyrus, right subcentral area, left dorsolateral prefrontal cortex, and precentral gyrus in RS-fMRI after chemotherapy. We found changes in the fornix and superior fronto-occipital fasciculus with DTI. There was a correlation between some cognitive function and MRI measurements in the correlation analysis, but it was not significant after false discovery rate (FDR) multiple testing corrections. The results indicate that RS-fMRI and DTI may be a prospective application for assessing chemotherapy related cognitive disorder.

## INTRODUCTION

Cognitive impairment related to chemotherapy, “chemo-brain”, has been reported in many cross-sectional and longitudinal studies by means of neuropsychologic assessments, magnetic resonance imaging (MRI), and positron emission tomography (PET). Some investigators report that cognitive impairment is not obvious comparing before and after chemotherapy results [1–3], some conclude that cognitive impairment exists and is reversible [1, 3, 4], while others think it is irreversible [5, 6]. In 2012, Heather S. L. Jim, et al. [7] argued from a meta-analysis that observed cognitive deficits in patients with breast cancer previously treated with chemotherapy are small in magnitude. However, in 2013 Gary Rodin et al.[8] reported that chemotherapy could induce cognitive impairment, and was worse than the estimation of previous studies. It is apparent that the understanding of “chemo-brain” needs more comprehensive research.

Many studies have used MRI to detect chemotherapy-related cognitive disorder. But when using fMRI, most choose task state functional MRI [9–11], although a few have used resting state functional MRI [11]. RS-fMRI is a reliable, non-invasive method for examining the intrinsic topology of large-scale brain networks [12], and it is based on blood oxygenation level dependent (BOLD) effect and can reflect the spontaneous neuronal activity [13]. It has been used to study neural and mental illness, and more evidence reveals a close relationship between cognitive function and RS-fMRI [14, 15]. Regional Homogeneity (ReHo), a model-driven method, measures the neural synchronization of a given voxel with its neighboring voxels by calculating Kendall's coefficient of concordance (KCC). It has been widely applied to study various neuropsychiatric diseases: mild cognitive impairment (MCI) and Alzheimer's disease (AD) [16], late-onset depression (LOD) [17], and schizophrenia [18].

Magnetic resonance diffusion tensor imaging (DTI) -a technique enabling the visualization and characterization of the white matter (WM) architecture via the self-diffusion of water molecules- allows us to study potential chemotherapy-induced changes in the WM [19]. Damage to WM structures may change quantitative DTI parameters, including fractional anisotropy (FA), which characterizes the degree of the directional preference of diffusion [20]. Chemotherapy-induced cerebral white matter impairment indicated by the DTI measure FA has been proven many times [21–23].

We combined RS-fMRI with DTI to detect chemotherapy-correlated cerebral changes from different perspectives. According to past relative RS-fMRI and DTI studies, we hypothesized that changes of ReHo and FA in some cerebral regions will occur after chemotherapy and that these changes will be associated with changes of performance in cognition.

## MATERIALS AND METHODS

### Patients

Thirty patients from Affiliated Union Hospital of Fujian Medical University were recruited. Nineteen women, age 43.1±8.8 years old, with early breast cancer who were scheduled to receive neoadjuvant or adjuvant chemotherapy (CT group). At the start of the study 13 women were premenopausal, five women were postmenopausal, and one patient's menstruation state was unclear because of a hysterectomy. To control for the effect of age, education and menstruation state, the controls were matched to the CT group on these factors as closely as possible. Eleven matched women, age 42.4±5.3 years old, participated in the study as the healthy control group (HC group). Seven women were premenopausal and four women were postmenopausal. These healthy controls all underwent mammary resections of benign neoplasms under general anesthesia: neoplasms such as fibroadenoma, sclerosing adenosis and hyperplasia. All were right-handed and had received at least nine years of standard education. Exclusion criteria included psychological disorders such as anxiety and depression, a history of other cancers, a history of nervous system damage (such as brain trauma, cerebral infarction, and cerebral hemorrhage), and drug or alcohol abuse. This study was approved by the Ethics Committee of the Affiliated Union Hospital of Fujian Medical University and the study was carried out in accordance with the Declaration of Helsinki. All subjects provided written informed consent for all of the procedures described in this report. Patient demographics and clinical characteristics are summarized in Table 1.

**Table 1:** Background and Clinical Characteristics of Patients and Controls

Characteristic	Patients Treated With Chemotherapy(CT) (n =19)		Healthy Controls (HC)(n=11)		Paired t-test	P
	Mean or Count	SD	Mean or Count	SD		
Age	43.1	8.8	42.4	5.3	0.252	0.803
Breast cancer stage						
I	6	N/A	N/A	N/A	N/A	N/A
II	11	N/A	N/A	N/A	N/A	N/A
III	2	N/A	N/A	N/A	N/A	N/A
Protocol of adjuvant chemotherapy						
3FET-3T	7	N/A	N/A	N/A	N/A	N/A
4EC-4T	7	N/A	N/A	N/A	N/A	N/A
4TC	3	N/A	N/A	N/A	N/A	N/A
6TEC	2	N/A	N/A	N/A	N/A	N/A
Test duration	118.6	32.2	118.7	23.8	-0.013	0.990
STAI at baseline						
S-AI	34.7	10.3	23.5	10.0	-2.93	0.007*
T-AI	27.8	9.1	21.8	8.2	-1.793	0.084
Years of education	13.5	3.6	12.0	2.9	1.194	0.242
menopause at baseline	13	N/A	7	N/A	N/A	N/A

* Statistically significant; N/A: not applicable; SD: standard deviation; 3FET-3T: three cycles fluorouracil, epirubicin and cyclophosphamide +three cycles paclitaxel; 4EC-4T: four cycles epirubicin, and cyclophosphamide +four cycles paclitaxel; 4TC: four cycles docetaxel and cyclophosphamide; STAI: State-Trait Anxiety Inventory; S-AI: State Anxiety Inventory: T-AI: Trait Anxiety Inventory.

### Chemotherapy

There were four kinds of chemotherapy regiments: 1) three cycles of 5-fluorouracil, epirubicin, and cyclophosphamide followed by three cycles of paclitaxel; 2) four cycles of epirubicin and cyclophosphamide followed by four cycles of paclitaxel; 3) four cycles of docetaxel and cyclophosphamide; 4) six cycles of paclitaxel, epirubicin, and cyclophosphamide. Two patients underwent neoadjuvant chemotherapy and finished treatments before surgery. The doses were standard according to NCCN clinical practice guidelines. The duration of chemotherapy was from 60 to 174 days. Adjuvant drugs included antacids (lansoprazole, pantoloc, and famotidine), antiemetics (dexamethasone, ondansetron, and ramosetron) and antiallergic drugs (dexamethasone). Granulocyte colony-stimulating factor was used when the complications included sever bone marrow suppression.

### Neuropsychologic assessment

A member of the research team interviewed patients and controls in a quiet room in the hospital. For the patients who received adjuvant chemotherapy and the controls, the first neuropsychologic assessment (t1) was two to three weeks after the operation and before chemotherapy. For the patients who received neoadjuvant chemotherapy, the first assessment was just before chemotherapy. The second assessment (t2) was two to three weeks after the chemotherapy for the patients receiving adjuvant therapy, and the second assessment was two to three weeks after the operation for the patients receiving neoadjuvant therapy. The controls’ second assessment was at a matched time.

The neuropsychologic assessments were performed on the same day as the MRI scan. The following neuropsychologic tests were administered:

The Functional Assessment of Cancer Therapy-Cognitive Function (FACT-COG) (Version 3) [24] is a self-assessment of cognition for patients receiving cancer therapy. It is a questionnaire including four domains: perceived cognitive impairments (CogPCI), impact of perceived impairments of quality of life (CogQOL), comments from others (CogOth), and perceived cognitive abilities (CogPCA). Items are summed to give scores for each domain and an overall QL score.

The Montreal Cognitive Assessment (MoCA) is a brief cognitive screening tool with high sensitivity and specificity [25]. It can evaluate several areas: visuospatial, memory, attention, and fluency [26].

The WAIS (III) Digit Symbol is a test evaluating processing speed [27]. The WAIS (III) Digit Span is a brief test that evaluates attention and includes two parts, digits forward and digits backward [21].

The Auditory Verbal Learning Test (AVLT) is used to assess memory ability [28]. This test contains three parts: short term delayed recall (AVLT1), long term delayed recall (AVLT2), and recognition (AVLT3).

The State-Trait Anxiety Inventory (STAI) [29] was used in our study to exclude anxiety disorders and assess the anxiety baseline of both the CT and HC groups.

The Beck Depression Inventory (BDI) [30] was used in our study to exclude depression disorder and assess the depression baseline of both CT and HC groups.

### Acquisition details for magnetic resonance imaging scans

All MRI imaging data came from the Radiology Department of the Affiliated Union Hospital of Fujian Medical University. MRI scans were performed on a MR750 3.0T scanner (General Electric Medical Systems, USA) with a high-resolution 8-channel head coil. Foam padding was used to limit the head motion and ear plugs were used to reduce scanner noise. Before RS-fMRI acquisition, all subjects were told to relax, and keep their eyes closed, but not fall asleep (confirmed after the scan). RS-fMRI data was collected axially by an echo-planar image (EPI) pulse sequence with 44 axial slices, thickness/gap = 3.2/0mm, matrix = 64×64, TR = 2000ms, TE = 29ms, flip angle = 90°, and FOV = 240×240mm. A total of 240 time points was obtained in 8 min. Diffusion-weighted imaging had the following parameters: Spin Echo-EPI, 20 diffusion weighting directions, matrix size = 128×128, repetition time = 8, 500ms, echo time = 86.4ms, field of view = 256×256mm, slice thickness = 68 slices, nex = 1, resolution = 2×2×2mm, b0 volumes number = 3, and b-value = 1, 000 s/mm^2^. High-resolution 3D-T1 (TR = 8.2ms, TE = 3.3ms, flip angle = 12°, thickness/gap = 1.0/0mm, FOV = 240×240mm, matrix = 256×256) and T2-FLAIR were also acquired. T2-FLAIR images were evaluated by two experienced radiologists. None of the subjects had obvious abnormalities in gross brain structure. The time of the examination was about 30 minutes.

### RS-MRI processing

All preprocessing was conducted using Statistical Parametric Mapping (SPM8, http://www.fil.ion.ucl.ac.uk/spm) and Data Processing Assistant for Resting-State fMRI (DPARSF2.2, http://www.restfmri.net) [31]. The first 10 time points were removed to obtain a stable resting state. The remaining 230 time points were processed by the following steps: 1) slice timing correction, all the remaining 230 volumes were corrected for the acquisition delay between slices, and aligned to the first image of each session for motion correction; 2) realignment, the images were first realigned to correct for head motions using the least-squares minimization; 3) normalization, unified segmentation using 3D-T1 images and spatial normalization using the deformation parameters; 4) smoothing, a Gaussian smoothing kernel with FWHM of 4×4×4mm^3^ was used to smooth the images to reduce noise and residual differences [13]; 5) time course de-trending and band-pass filtering (0.01-0.08Hz) was applied to remove physiological and high frequency noise; 6) 6 head motion parameters and signals for the WM and cerebrospinal fluid (CSF) were regressed out. The subjects whose head motions exceed 2mm of movement or two degrees in any direction should be excluded.

The ReHo [32], assuming that a given voxel is temporally similar to that of its neighbors by calculating KCC, can effectively evaluate the resting-state brain activity. Furthermore, the test-retest reliability of ReHo was established recently by Zuo X.N. et al.[33]. By calculating KCC at each voxel between the time series of the voxel and those of its 26 neighboring voxels, an individual ReHo image [32] was generated for each subject within a whole-brain mask. The mask was provided by DPARSF, and excluded non-brain areas. Then each subject's ReHo image was standardized by their own mean KCC within the mask, using the same whole brain mask [32]. In this step, the KCC value of each voxel was divided by the mean KCC within the mask in each individual ReHo map. Then, a Gaussian smoothing kernel with a FWHM of 4×4×4mm^3^ was used to smooth the images to reduce noise and residual differences.

The REST1.8 (http://www.restfmri.net) [34] was used for statistical analysis. Lilliefors goodness-of-fit test of all subjects was employed to assess whether all subjects were from a normal distribution. Then we also compared the ReHo results between t1 and t2 by performing voxel-based paired t tests in both groups separately. The *P* < 0.05 was deemed significant for the AlphaSim correction (the parameters used in the AlphaSim program: cluster size >1458mm^3^, spatial smoothness = 4, rmm = 4, a whole-brain mask provided by DPARSF). Based on the paired t test findings, the abnormal ReHo brain regions were identified as regions of interest (ROIs), the mean zReHo value of each ROI for all the subjects was extracted.

### DTI processing

We used a pipeline tool called PANDA (http://www.nitrc.org/projects/panda/) [35] to process data by the following steps:

1) Converting format: Dicom files of DTI raw data were converted into Nifti format during this step. The dcm2nii tool embedded in MRIcron accomplished this task.

2) Estimating the brain mask: This step yielded the brain mask by using the bet command of FSL (FMRIB Software Library v5.0) [36]. The brain mask was required for the subsequent processing steps. Here, the b0 image without diffusion weighting was used for the estimation.

3) Cropping the raw images: To reduce the memory cost and speed up the processing in subsequent steps, we cut off the non-brain space in the raw images to reduce the image size. The acquired brain mask was used to determine the borders of the brain along the three dimensions. The fslroi command of FSL was then applied to remove the non-brain spaces.

4) Correcting for the eddy-current effect: Eddy-current induced distortion of diffusion weighted images (DWI), as well as the simple head-motion during scanning, can be corrected by registering the DWI images to the b0 image with an affine transformation. We used the flirt command of FSL to achieve this. Finally, the gradient direction of each DWI volume was rotated according to the resultant affine transformations.

5) Calculating diffusion tensor (DT) metrics: This step involves a voxel-wise calculation of the tensor matrix and the DT metrics, including FA. The dtifit command of FSL was applied, producing diffusion metrics that are ready for statistical analysis.

6) Normalizing: To allow for comparison across subjects, location correspondence has to be established. To end this, registration of all the individual images to a standardized template are always applied. Here, we conducted non-linear registered individual FA images of native space to the FA template in the MNI space by executing the fnirt command of FSL. The resultant warping transformations were then used to resample the images of the diffusion metrics (FA) into the MNI space with a customized spatial resolution (1×1×1mm). This resampling step was implemented by the applywarp command of FSL.

7) Output for atlas-based analysis: In addition to the voxel-based method of analysis, diffusion metrics can be analyzed at the level of ROIs, which may provide better statistical sensitivity. These WM atlases in the standard space allow for parcellation of the entire WM into multiple ROIs, each representing a labeled region in the atlas. These ROIs were anatomically independent. We calculated the regional diffusion metrics FA by averaging the values within each region of the ICBM-DTI-81WM labels atlas [37]. Then these results (saved as excel files) was statistically analyzed with SPSS 13.0.

### Clinical assessment statistics

All statistics were performed with IBM SPSS 13.0 (SPSS, Chicago, IL). A two samples t test was used to contrast the two groups’ clinical characteristics including age, education, and STAI at baseline. A chi-square test was used to contrast the menstruation state. The BDI scores were not distributed normally, so we used the Mann-Whitney U test to contrast the two groups’ baseline BDI and the results revealed that the BDI score of the CT group was significantly higher (*P* < 0.05). Because the groups differed significantly in terms of S-AI anxiety and BDI depression scores at baseline, we included both variables as covariates. Analysis of covariance was used to assess the neuropsychologic and MRI data differences between the groups at baseline with both BDI and S-AI as covariates. Paired t tests were used to assess changes in the neuropsychologic test performance and MRI data between t1 and t2 within the separate groups. FDR multiple corrections were applied in the multiple comparisons of FA values. Statistical significance was assessed at *P* < 0.05. To assess the interaction of both time and group in neuropsychologic assessments, we used one-way repeated measures ANOVA with time as a within-subject factor, group as a between subject factor, and BDI and S-AI as the covariates.

### Correlation analysis

To study the effect of cognitive impairment on intrinsic cerebral activity and WM integrity, we carried out a correlation analysis between the longitudinal change in all objective test performance and change in ReHo and FA values of the ROIs that survived the paired t tests separately (change was calculated as t2 minus t1). For the different baseline of S-AI anxiety and BDI depression between the two groups, we used partial correlation analysis to assess their correlation with S-AI and BDI as covariates. We also made a correlation between the ReHo and FA values. Statistical significance was assessed at *P* < 0.05 with FDR corrections for comparing multiple groups.

## RESULTS

A total of forty participants were initially recruited for our study. Five patients were excluded because of head motion at t1 or t2, four patients did not complete the follow-up MRI assessment, and one patient was excluded because of an unknown intracranial occupying lesion. Therefore, the final sample size for the longitudinal analysis was 19 patients who received chemotherapy and 11 healthy controls.

### Participant demographic and clinical data

Participant demographic and medical information is summarized in Table 1. Groups did not differ significantly in terms of age, education, and menstruation state at baseline (Chi-square results of menstruation state: χ^2^ = 0.07, *P* = 0.789). The Mann-Whitney Test of BDI showed that the mean rank of the CT and HC groups were 20.5 and 6.8, the sum of ranks were 390.0 and 75.0. The value from the Mann-Whitney U test was 9.0 (*P* < 0.0001). That means that the level of depression in the CT group was higher than in the HC group. In the CT group, all premenopausal patients before chemotherapy were menopausal at t2. Only one patient's menstruation state was unclear due to history of hysterectomy. Menopause here refers to the pause of regular menses. The menstruation state of the HC group was unchanged.

### Neuropsychologic assessment

After controlling for the depression and anxiety scores, there was no significant difference between patients with cancer and healthy controls in the different cognitive domains cognitive functioning at baseline. Paired t tests showed that the chemotherapy-treated group performed significantly worse after chemotherapy on the memory test (AVLT, especially recognition) and self-assessment on cognition (FACT-COG) when compared with the baseline (*P* < 0.05). The healthy controls’ self-assessment on cognition increased (*P* < 0.05). In addition, the repeated measures ANOVA also revealed significant interactions between groups and a performance change over time for the memory test (especially recognition) and self-assessment on cognition (*P* < 0.05)(Table 2).

**Table 2:** Summary of neuropsychologic assessments

Tests	Patients Treated With Chemotherapy (CT)(*n* =19)					Healthy Controls (HC)(*n*=11)					Group Time Interaction With Repeated Measures ANOVA	
	t1		t2		Paired t-test	t1		t2		Paired t-test	F	*P*
	mean	SD	mean	SD		mean	SD	mean	SD			
AVLT	31.42	8.051	26.53	8.650	0.016*	29.64	11.138	30.00	11.533	0.542	4.554	0.042*
short term	10.53	1.982	10.00	2.582	0.340	11.55	2.659	12.00	2.966	0.138	1.746	0.197
long term	8.74	3.070	8.11	3.430	0.383	8.82	3.710	9.00	3.899	0.506	0.725	0.402
recognition	12.16	3.516	8.42	3.761	0.001*	9.27	5.287	9.00	5.177	0.341	8.076	0.008*
Digit Symbol	52.84	15.770	51.26	15.202	0.450	54.64	14.080	55.55	14.123	0.264	0.809	0.376
Digit Span forward	6.05	1.353	5.89	1.629	0.546	6.36	1.286	6.73	1.191	0.167	1.825	0.188
backward	5.05	1.026	5.11	1.761	0.848	5.91	1.514	5.73	1.272	0.341	0.375	0.545
MOCA	25.95	3.659	25.58	3.878	0.414	26.91	2.982	27.00	2.793	0.724	0.561	0.460
FACT-COG	106.21	23.220	89.00	25.126	0.003*	117.18	7.808	119.64	7.103	0.036*	8.896	0.006*
CogPCI	57.16	13.937	46.11	15.613	0.001*	63.82	6.369	64.91	5.431	0.210	10.094	0.004*
CogQOL	12.21	4.650	12.05	4.288	0.888	14.55	2.544	15.73	0.647	0.168	0.717	0.404
CogOth	14.26	3.177	12.21	4.276	0.070	15.09	1.868	15.45	1.293	0.341	2.823	0.104
CogPCA	22.58	4.598	18.63	6.335	0.019*	23.73	3.977	23.55	3.698	0.640	3.354	0.078

* Statistically significant; ANOVA: analysis of variance; AVLT: Auditory Verbal Learning Test; MOCA: Montreal Cognitive Assessment; FACT-COG: Functional Assessment of Cancer Therapy-Cognitive Function; CogPCI: perceived cognitive impairments; CogQOL: impact of perceived impairments on quality of life; CogOth: comments from others; CogPCA: perceived cognitive abilities; SD: standard deviation.

### RS-fMRI and DTI results

**Figure 1:**
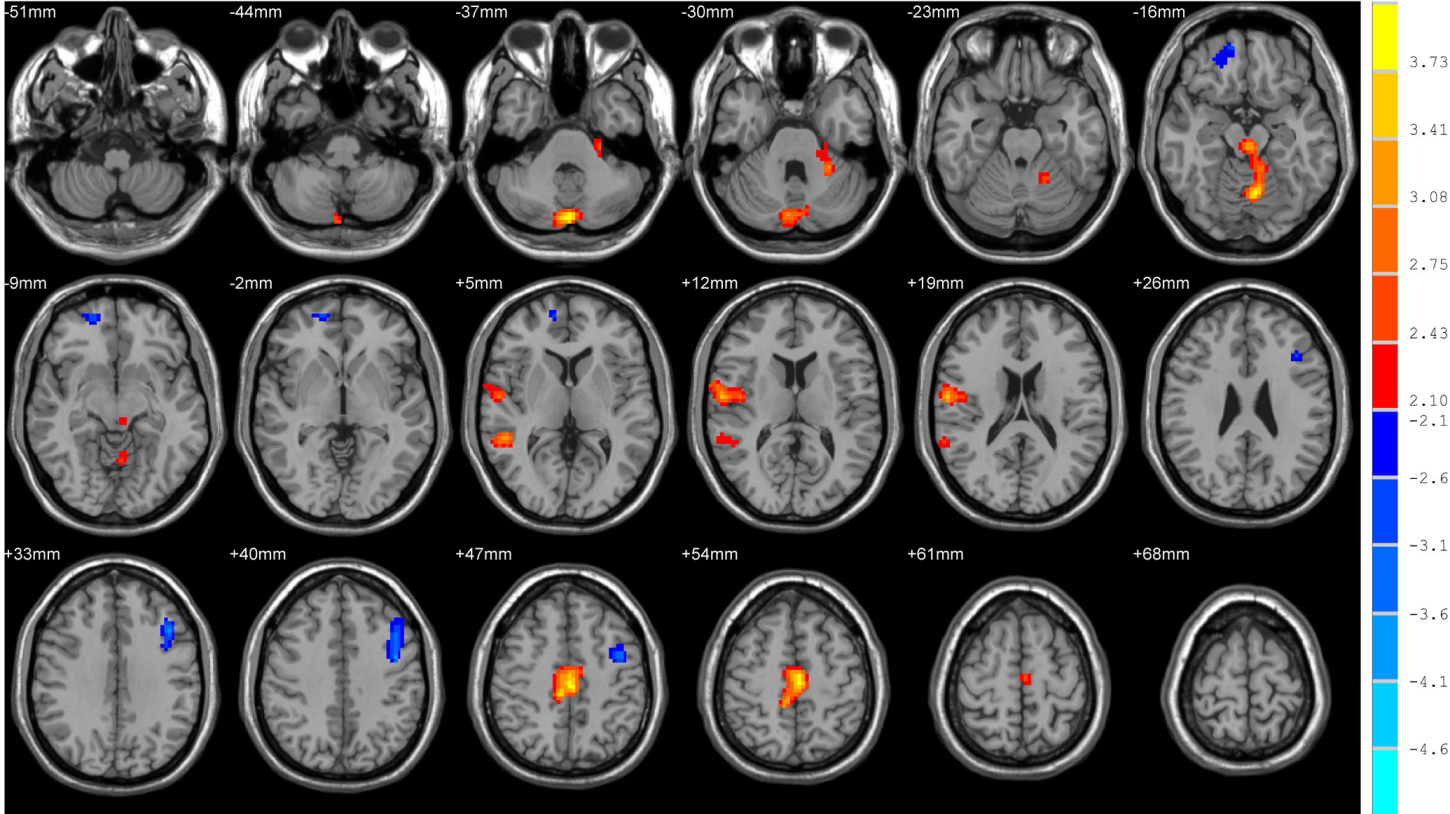
Brain areas with significant changes of ReHo values after chemotherapy in paired t tests Warm and cool colors indicate significantly decreased and increased ReHo values at *P* < 0.05 (AlphaSim corrected, cluster size>1458 mm3, spatial smoothness = 4, rmm = 4).

After controlling for the depression and anxiety scores, there was no significant difference between the two groups for the ReHo and FA of the ROIs at baseline. The Lilliefors goodness-of-fit test of all RS-fMRI data showed that the P-value of most voxels was greater than 0.05, we consider that the sample were normally distributed. In the HC group, we found no significant difference of ReHo values by performing voxel-based paired t tests between t1 and t2. In the CT group voxel-based paired t tests, we found significant increases of ReHo in the posterior lobe of the cerebellum and the anterior lobe of the cerebellum, and significant decreases in the right orbitofrontal area, right middle and superior temporal gyrus, right subcentral area (between the insula and post/precentral gyrus), and the left dorsolateral prefrontal cortex and precentral gynus (Table 3 and Figure 1). For the DTI analysis, FA values have been compared in predefined regions according to the ICBM DTI-81 atlas. This atlas contains 50 regions, and differences were found in 3 out of the 50 regions in the CT group without FDR corrections. The regions were the fornix, the left superior fronto-occipital fasciculus (SFOF), and the right SFOF (*P* < 0.05) (Table 4). However the corrections results showed that they were not significant after FDR corrections. In the HC group, we found no significant difference of FA values when performing paired t tests between t1 and t2.

**Table 3:** ReHo paired t test for patients treated with chemotherapy

Region	BA	MNI coordinate			Z	Cluster size	P	t	Mean zReho	
		X	Y	Z					t1 t2	
Posterior lobe of cerebellum	/	0	-84	-36	4.0585	132	0.000*	-5.185	0.1522	0.4676
Anterior lobe of cerebellum	/	-6	-63	-15	3.7676	295	0.000*	-4.472	0.2730	0.5273
Right orbitofrontal area	11	24	45	-18	-2.9596	107	0.000*	5.194	-0.0722	-0.3190
Right middle and superior temporal gyrus	21, 22	51	-42	6	3.3071	84	0.000*	6.252	-0.4044	-0.6641
Right subcentral area	43	60	-9	15	3.7736	181	0.001*	4.058	0.3506	0.0474
Left dorsolateral prefrontal cortex	9	-42	-3	42	-5.1767	252	0.000*	4.793	-0.0802	-0.3795
Precentral gyrus	6	-3	-24	51	4.0519	287	0.000*	4.929	0.1819	-0.1204

* Statistically significant; BA: Brodmann area.

**Table 4:** FA paired t test for patients treated with chemotherapy

Region	mean FA		Paired t	*p*
	t1	t2		
Fornix	0.499	0.489	2.293	0.035*
SFOF-R	0.478	0.463	2.268	0.037*
SFOF-L	0.476	0.461	2.155	0.046*

* Statistically significant; SFOF-R: right superior fronto-occipital fasciculus; SFOF-L: left superior fronto-occipital fasciculus; FA: fractional anisotropy.

### Correlation analysis

In partial correlation analysis (Table 5), performance changes in attention correlated with ReHo changes in the left dorsolateral prefrontal cortex. Changes in short term memory correlated with ReHo changes in the precentral gyrus with BDI depression and S-AI anxiety as covariates (*P* < 0.05). There was no significant correlation after FDR multiple testing corrections. There was no correlation between the longitudinal change in test performance that survived the paired t tests and change in FA values of the ROIs that survived the paired t tests. There was also no significant correlation between the ReHo and FA values.

**Table 5:** Partial correlation analysis between differences of ReHo values and differences of neuropsychologic assessments in patients treated with chemotherapy

Region	Domain (Test)	Correlation Coefficient	*P*
Left dorsolateral prefrontal cortex	Attention(DSp2)	0.486	0.048
Precentral gyrus	Memory(AVLT1)	-0.502	0.040

ReHo: regional homogeneity; P was not corrected for multiple comparisons; DSp2: backward part of Digit Span; AVLT: Auditory Verbal Learning Test; AVLT1: short term delayed recall of AVLT.

## DISCUSSION

There have been a number of other studies that have examined neuropsychologic tests and brain imaging in breast cancer patients before and after chemotherapy. But the evidence is mixed regarding “chemo-brain”. Some considered observed cognitive deficits in patients with breast cancer treated with chemotherapy are unconspicuous [1–3, 7], the other studies showed that chemotherapy leads to cognitive disorder even there exists the risk of underestimation [4–6, 8]. An observational longitudinal study indicated that chemotherapy disorder focused on verbal and working memory, the chemotherapy patients were more likely to show cognitive decline than controls (OR 2.25) [4]. Some study even indicated the persistence of cognitive disorder 5 years after completion of chemotherapy [5]. Possible reasons why the studies results are so different may attribute to different design of experiments, follow-up time, monitoring point in time, control group setting, sample size, chemotherapy regimens and other unidentified factors.

Most studies showed that chemotherapy induces cognitive disorders, such as attention [38]^,^ [39], executive ability [38]^,^ [39], learning ability [39], memory [21, 38], verbal ability [7]^,^ [21] and visuospatial ability [7]. In this study, paired t tests revealed that memory, especially recognition memory, declined significantly after chemotherapy while that of the healthy controls stayed the same. We speculate that the chemotherapeutic damage focuses on memory function. A report which studied chemotherapy-related cognition impairment and brain white matter changes in breast cancer patients also found decreased memory after treatment [21]. And this point has been confirmed in many other similar reports. Part CogPCI and CogPCA of the FACT-COG questionnaire, which reflect subjective cognition disorders, also reflected a decline in the cancer patients. Clinical experience is consistent, as most patients complained of memory decline after chemotherapy. Therefore, in our opinion chemotherapy may induce damage to cognition, mainly to memory.

Even though there was no significant change of objective cognitive tests in the HC group, the values of the subjective FACO-COG test increased. Does it mean that the cognition of the HC group began to be restored at t2? A significant proportion of people show postoperative cognitive dysfunction (POCD) in early weeks after major surgery under general anesthesia and the incidences have been reported to be between 25% one week after surgery and 10% after three months [40]. That means the cognition of some of the patients may recover partly or totally three months after surgery. The mean test duration of the HC group in our study is 118.7 days. We speculate that the higher FACT-COG values of the HC group can be due to the recovering of POCD.

Significantly changed cerebral regions after chemotherapy in most relative literature covered the frontal lobe [10, 21, 41] whose function involves attention, executive ability, and learning ability and the hippocampal gyrus [11]^,^ [38] whose function involves memory ability. The frontal lobe was also a meaningful cerebral region in our results, with decreases in ReHo in the right orbitofrontal area and left dorsolateral prefrontal cortex. In addition, significant decreases of ReHo in the right orbitofrontal area, right middle/superior temporal gyrus, and left dorsolateral prefrontal cortex were close to the results of studies by Jennifer Bruno et al. who found the nodal degree and number of hubs decreased in the regions of the dorsolateral prefrontal, orbitofrontal, and middle temporal regions when studying altered resting state functional brain network topology in breast cancer patients. These cerebral regions may correspond to the deficits in executive function, memory and learning, and regulation of emotions [11].

We found that the FA values of fornix declined after chemotherapy (Table 4). Recent work has shown that the fornix is associated with memory control [42, 43] and has been shown to be a predictor of earliest cognitive decline [43]. There was an apparent decrease measured in the memory test, even though we did not find a significant correlation between FA changes of the fornix and memory test in our results. Based on the sensitivity of the fornix for cognitive impairment, changes of fornix microstructure in the DTI may also be a predictor of chemotherapy related cognitive disorders.

We detected a decrease of FA in the superior fronto-occipital fasciculus (SFOF). The SFOF is a part of a widely distributed visuospatial attention network [44] and is closely related with the superior longitudinal fasciculus (SLF). The SFOF overlaps with branches of the SLF and probably represents an “occipital extension” of the SLF [45]. Besides, the SLF, a long association tract integrating frontal and parietal association cortices [21], is known to be correlated with processing speed [46] and memory [47]. Sabine Deprez et al. found the FA values of DTI in the SLF also decreased after breast cancer chemotherapy, which provided direct evidence of the impact of chemotherapy on brain white matter [21].

We found the cognitive function associated with some ROI was inconsistent with corresponding brain regions as was our previous understanding in the correlation analysis of our results (Table 5). For example, the precentral gyrus is known to be associated with physical movement, but our results pointed to the short-term memory (AVLT test). In addition, the result suggested a negative and medium correlation between them. Even though these unconventional results were insignificant after FDR correction, we should not rule out the possibility of the correlation because of the lack of a sufficiently large sample size. More work is needed to verify the relationship between these cognitive domains and cerebral areas.

Some limitations of this study should be mentioned. First, the sample size was small and we could not perform a hierarchical analysis, such as considering menopausal status and chemotherapy. We should also consider that chemotherapy itself works differently on cognition depending on the dose, the number of cycles, and auxiliary medicines. Sanne B. Schagen et al. found that more patients treated with high-dose chemotherapy than patients treated with standard-dose chemotherapy showed a decline in cognitive performance compared with healthy control subjects [48]. That is to say, there is perhaps a dose-effect relationship between chemotherapy and cognition. The test duration, which was just the evaluation interval between t1 and t2, spanned from 60 to 174 days. This heterogeneity may have an effect on cognition assessment. There are some auxiliary medicines, such as an antiemetic, that are believed to be active in the central nervous system and to affect cognition [49] and they could also be an interfering factor. Second, our cognitive testing was not assessed by a licensed clinical neuropsychologist so there may be examiner bias. At last, we did not include a non-chemotherapy-exposed breast cancer control group so we cannot separate the effect of chemotherapy and the direct effect of cancer itself. Compared with the study of cognitive impairment induced by chemotherapy, the effect of the tumor on cognition has been relatively neglected. Some have studies have shown that pretreatment cognitive impairment exists for the breast cancer patients [50]^,^ [51, 52]. The results support the opinion that breast cancer itself can induce cognitive impairment. Recently, Kerstin Hermelink et al. carried out a large controlled study which showed that breast cancer patients may show limited cognitive impairment that seems to be largely caused by cancer-related post-traumatic stress prior to any treatment (including chemotherapy and surgery) [53]. We need a larger sample and longer follow-up time to implement further study to identify what are the specific and independent effects on cognition disorders in patients receiving cancer treatment.

In the past relevant studies, the time points for cognitive or imaging assessments evaluation after chemotherapy were various; and included 3 weeks [39], 1 month [10], 6 months [3, 48], 1 year [10], 18 months [48], 5years [11] and 10 years [54–56]. Some studies assessed the cognition at different time points to study the shifting trend of chemotherapy related cognitive disorders. We plan to design additional follow-up research to understand longitudinal change in cognitive function after treatment completion. But the control of confounding factors will become more difficult with the extension of the follow-up time. Factors such as accelerated aging of the brain which may play a part in chemotherapy related cognitive disorders five years or longer after treatment [11] and practice effect will need to be considered. Chemotherapy induced cognitive impairment may also be associated with genetic vulnerability factors: the genes coding apolipoprotein E (APOE) and catechol-O-methyltransferase (COMT) have been suggested to be variables [8]. We also collected serum samples from the subjects in synchronization with the neuropsychologic assessments and RS-fMRI scan and plan to carry out a subsequent serological study.

Even though we did not find a correlation between the neuropsychologic assessments and MRI data, RS-fMRI and DTI have the potential to detect the chemotherapy related cognitive disorder because of their sensitivity. Because there is no need for injection of radioactive drugs and performing tasks, RS-fMRI and DTI are more convenient and repeatable compared with the task state fMRI and PET. Therefore, RS-fMRI and DTI have potential application in assessing chemotherapy related cognitive disorders.
